# Oxidative Stress and Antioxidant Status in Adult Patients with Transfusion-Dependent Thalassemia: Correlation with Demographic, Laboratory, and Clinical Biomarkers

**DOI:** 10.3390/antiox13040446

**Published:** 2024-04-10

**Authors:** Antonella Meloni, Laura Pistoia, Anna Spasiano, Antonella Cossu, Tommaso Casini, Antonella Massa, Sergio Bagnato, Maria Caterina Putti, Silvia Maffei, Vincenzo Positano, Alessia Pepe, Filippo Cademartiri, Cristina Vassalle

**Affiliations:** 1 Bioengineering Unit, Fondazione G. Monasterio CNR-Regione Toscana, 56124 Pisa, Italy; antonella.meloni@ftgm.it (A.M.); positano@ftgm.it (V.P.); 2 Department of Radiology, Fondazione G. Monasterio CNR-Regione Toscana, 56124 Pisa, Italy; laura.pistoia@ftgm.it (L.P.); fcademartiri@ftgm.it (F.C.); 3 Unità Operativa Complessa Ricerca Clinica, Fondazione G. Monasterio CNR-Regione Toscana, 56124 Pisa, Italy; 4 Unità Operativa Semplice, Dipartimentale Malattie Rare del Globulo Rosso, Azienda Ospedaliera di Rilievo Nazionale “A. Cardarelli”, 80131 Napoli, Italy; spasiano.anna@tiscali.it; 5 Ambulatorio Trasfusionale—Servizio Immunoematologia e Medicina Trasfusionale, Dipartimento dei Servizi, Presidio Ospedaliero “San Francesco”, 08100 Nuoro, Italy; a.cossu.1@aslnuoro.it; 6 Oncologia, Ematologia e Trapianto di Cellule Staminali Emopoietiche, Meyer Children’s Hospital IRCCS, 50139 Firenze, Italy; tommaso.casini@meyer.it; 7 Servizio Trasfusionale, Ospedale “Giovanni Paolo II”, 07026 Olbia, Italy; antonella.massa@aslgallura.it; 8 Ematologia Microcitemia, Ospedale San Giovanni di Dio—ASP Crotone, 88900 Crotone, Italy; krthal@libero.it; 9 Dipartimento della Salute della Donna e del Bambino, Clinica di Emato-Oncologia Pediatrica, Azienda Ospedaliero, Università di Padova, 35128 Padova, Italy; mariacaterina.putti@unipd.it; 10 Cardiovascular and Gynaecological Endocrinology Unit, Fondazione G. Monasterio CNR-Regione Toscana, 56124 Pisa, Italy; silvia.maffei@ftgm.it; 11 Institute of Radiology, University of Padua, 35128 Padova, Italy; alessia.pepe@unipd.it; 12 Medicina di Laboratorio, Fondazione G. Monasterio CNR-Regione Toscana, 56124 Pisa, Italy

**Keywords:** thalassemia major, oxidative stress, magnetic resonance imaging, iron overload, OXY-adsorbent assay, d-ROM test

## Abstract

Iron overload in beta transfusion-dependent thalassemia (β-TDT) may provoke oxidative stress and reduction of the antioxidant defenses, with serious consequences for the disease course and complications. The present study evaluated the oxidant/antioxidant status of β-TDT patients and its correlation with demographic, clinical, laboratory, and instrumental biomarkers. The OXY-adsorbent assay and the d-ROMs (Diacron, Grosseto, Italy) were evaluated in 58 β-TDT patients (mean age: 37.55 ± 7.83 years, 28 females) enrolled in the Extension-Myocardial Iron Overload in Thalassemia Network. Iron overload was quantified with R2* magnetic resonance imaging. Mean OXY was 323.75 ± 113.19 μmol HClO/mL and 39 (67.2%) patients showed a decreased OXY-Adsorbent level (<350 μmol HClO/mL), of whom 22 (37.9%) showed severely reduced levels. Mean d-ROMs was 305.12 ± 62.19 UA; 12 (20.7%) patients showed oxidative stress, and 4 (6.9%) elevated oxidative stress. OXY showed a significant negative correlation with global and segmental cardiac iron levels. D-ROMs levels significantly correlated with markers of cardiovascular risk (aging, glycemia, and N-terminal pro-B-type natriuretic peptide). Antioxidant depletion is frequent in β-TDT patients, where OXY might serve as additive biomarker to assess heart iron status, whereas the d-ROMs might be helpful to assess the cardiovascular risk burden.

## 1. Introduction

Thalassemia is an inherited hemoglobinopathy responsible for the impaired production of alpha (α-thalassemia) or beta (β-thalassemia) globin chains that results in non-functional hemoglobin, damaged erythrocytes, ineffective erythropoiesis, and anemia [1,2,3,4]. According to the World Health Organization (WHO), 40,000 infants with thalassemia are born annually worldwide, the majority of these having β-thalassemia [5]. Moreover, migration has increased the prevalence of the disease in areas where it was previously thought to be rare [6,7], continuing to keep this disease a major serious sanitary issue.

Historically, depending on the severity of the disease phenotype and the involved genetic mutations, β-thalassemia has been classified into three forms: minor, intermedia, and major [4,8]. Thalassemia minor, also known as thalassemia trait, is the mildest form of the disease, while β-thalassemia major (TM) is the most severe form, with affected patients typically presenting early in life with severe anemia. Thalassemia intermedia (TI) is a disease of intermediate severity. Transfusion therapy continues to be the primary treatment for severe types of thalassemia, with frequency and amount of needed transfusions providing an indirect measure of the severity of the underlying condition [9]. So, today, for management purposes, the thalassemia patients are commonly grouped into transfusion dependent (TDT) or non-transfusion-dependent (NTDT) [10]. The term (TDT) is mainly applied to individuals with β-TM who are unable to produce sufficient amounts of hemoglobin to survive without blood transfusions [11]. The other side of the coin is that regular transfusions markedly increase the accumulation of iron in the body [12,13]. Consequently, patients with TDT must adhere to lifelong chelation therapy regimens to mitigate the adverse effects associated with iron overload, thereby reducing the risk of complications and mortality [14,15,16].

Despite the advancements in iron chelation, secondary iron overload is still a major issue in β-TDT [17]. Consequently, β-TDT patients may incur in oxidative stress with overproduction of reactive oxygen species (ROS), which can lead to growth lag, delayed sexual development, and adverse consequences also for liver, heart, and endocrine system functioning [18,19]. Moreover, an elevated oxidative stress, which results from a shift in the balance between ROS and the antioxidant defense system, has been demonstrated as a key determinant in the pathogenesis of different chronic-degenerative diseases related to aging, including cardiovascular disease and Type 2 diabetes [20], which represent the more frequent and critical complications in β-TDT patients [21,22,23]. This issue is of growing importance also in view to the extended life expectancy of TDT patients, [24] with more than half of them expected to survive beyond the age of 50.

This study aimed to assess the oxidative stress status in the serum of well-treated β-TDT patients by evaluating the levels of the Diacron OXY-adsorbent assay (as an index of the total antioxidant capacity) and of the reactive oxygen metabolites (as an index of the oxidant counterpart), and to evaluate the relationship of these biomarkers with demographic, clinical, laboratory, and instrumental parameters.

## 2. Materials and Methods

### 2.1. Study Population

The Extension-Myocardial Iron Overload in Thalassemia (E-MIOT) project is an Italian collaborative Network involving 66 thalassemia centers and 15 validated magnetic resonance imaging (MRI) sites [25]. These centers are interconnected through a web-based database, which serves as a centralized repository for gathering and managing a wide range of clinical, anamnestic, laboratory, and instrumental data. The inclusion criteria of the E-MIOT project are: (1) both males and females, spanning a wide range of age groups, diagnosed with either thalassemia or sickle cell disease and necessitating the assessment of organ iron levels through MRI; (2) written informed consent for participation in the study; (3) written agreement for the utilization or disclosure of protected health information; and (4) no contraindications to MRI scanning.

In the years 2015 and 2016, as part of a pilot study aimed at assessing the relationship between osteoporosis and cardiovascular diseases in thalassemia, all adult β-TDT patients attending the reference MRI center of the E-MIOT Network (Fondazione G. Monasterio CNR-Regione Toscana (FTGM), Pisa, Italy) were requested to undergo, on the same day of the MRI scan, a blood test for the assessment of various parameters related to both bone and cardiovascular health. In a small subgroup of patients participating to the project (N = 58), the biochemical assessment of oxidative stress biomarkers was also performed.

The E-MIOT and the collateral projects received approval from the Ethics Committee of Area Vasta Nord Ovest (CEAVNO). The study was conducted in accordance with the principles outlined in the Declaration of Helsinki. Written informed consent was obtained from all patients before enrollment.

### 2.2. Biochemical Analysis

Serum pre-transfusion hemoglobin, ferritin, aspartate aminotransferase (AST), alanine aminotransferase (ALT), total triglycerides, total cholesterol, and high-density lipoprotein (HDL) were determined at the thalassemia centers where the patients were treated using commercially available kits. The assessments of hemoglobin, ferritin, and liver transaminases were performed at least four times per year, and for each patient, a single value was obtained by averaging the multiple measurements.

To assess the disturbances of glucose metabolism, patients non already diagnosed with diabetes performed an oral glucose tolerance test (OGTT) within three months from the MRI. All patients were required to fast for the entire night, and a blood sample was drawn to assess fasting glucose and insulin. Patients were given 1.75 g/kg (maximum dose of 75 g) of glucose solution and glucose and insulin were measured at 60 and 120 min.

At FTGM, blood samples were collected after 8 h of fasting and were handled and analyzed in the Medicine Laboratory under strictly standardized conditions and in agreement with the manufacturers’ recommendations.

Samples for N-terminal Pro–B-Type Natriuretic Peptide (NT-proBNP) assessment were kept on ice, immediately centrifuged, and stored at −80 °C until analysis. NT-proBNP was measured on the Cobas e411 analyzer (Roche Diagnostics, Basilea, Switzerland) using the proBNP II kit, reported to have a limit of detection at 5 ng/L and a threshold value of 125 ng/L (inter-assay coefficient of variation 4.2% at 44 ng/L, 2.4% at 126 ng/L, and 1.3% at 2410 ng/L).

The total antioxidant capacity was measured using a spectrophotometric assay (OXY-adsorbent assay, Diacron, Grosseto, Italy) in blood samples. This method relies on the ability of a high dose of hypochlorous acid (HClO) to oxidize physiological antioxidants present in the serum sample, such as uric acid, glutathione, thiol groups, vitamins, glutathione peroxidase, superoxide dismutase, catalase, and others [26]. After 10 min incubation, residual HClO undergoes a reaction with an alkyl-substituted aromatic amine (A-NH2, solubilized in a chromogenic mixture); amine is oxidized by HClO, producing a colored product, which is photometrically measured. The concentration of the colored complex formed is directly proportional to the concentration of HClO in the sample and inversely proportional to the antioxidant capacity. The results are typically expressed as the amount of HClO consumed per milliliter of sample (μmol HClO/mL). Intra- and inter-assay coefficients of variation at each level tested on 10 aliquots of fresh samples and on 10 aliquots of frozen samples were always lower than 5.5% [27].

Reactive oxygen species were evaluated in serum using a kinetic spectrophotometric assay (d-ROMs test, Diacron, Grosseto, Italy). This test exploits the ability of hydroperoxides to generate free radicals in the presence of transition metals, such as iron and copper, acting as catalyzers [26,28,29]. When these free radicals interact with a properly buffered chromogenic substance, they form a colored complex. This complex can be quantitatively measured using photometric techniques, with the maximum peak absorbance typically occurring at 505 nm. The intensity of the color is proportional to the peroxide concentration. The test result is displayed as an arbitrary unit (1 UA corresponding to the color development caused by a H_2_O_2_ solution at a concentration of 0.08%).

### 2.3. Magnetic Resonance Imaging

MRI scanning was performed within one week before a regularly scheduled blood transfusion using a 1.5 T scanner (Signa Excite or Artist, GE Healthcare, Milwaukee, WI, USA) and a 30-element cardiac phased-array receiver surface coil. All images were acquired with breath-holding and ECG gating.

The T2* technique was used for iron overload assessment. A mid-transverse hepatic slice, five or more axial slices including the whole pancreas, and three parallel short-axis views (basal, medium, and distal) of the left ventricle (LV) were acquired using multi-echo gradient echo sequences (10 echo times with an echo spacing of 2.26 ms). Image analysis was conducted using a custom-written, previously validated software (HIPPO MIOT^®^ Version 1.0, Consiglio Nazionale delle Ricerche and Fondazione Toscana Gabriele Monasterio, Pisa, Italy) designed to provide the T2* values for specific anatomical regions of interest (ROIs). All pixels within the ROI were averaged together, and this averaged decay curve was fit to an appropriate decay model (single exponential with a variable offset model for liver and pancreas and single exponential with truncation for the heart). The extracted T2* values were converted into R2* values, directly proportional to iron levels, using the formula: R2* = 1000/T2* [30]. Hepatic R2* values were calculated in a ROI of standard dimension drawn away from blood vessels and other sources of artifacts and were converted into liver iron concentration (LIC) values [30]. Three small ROIs were manually delineated over the pancreatic head, body, and tail, covering the parenchymal tissue and staying away from blood vessels or ducts and areas affected by susceptibility artifacts from intraluminal gas in the stomach or colon [31]. The global pancreatic R2* value was determined by averaging the R2* values from the three regions. The myocardial R2* distribution was mapped into a 16-segment LV model, in line with the American Heart Association/American College of Cardiology standardized segmentation model [32]. The global heart R2* value was the mean of all segmental R2* values. 

For the assessment of the cardiac dimensions and function, balanced steady-state free precession (SSFP) cine images in long axis (two-chamber, three-chamber, and four-chamber views) and short axis orientations (8 mm slice thickness, without gaps) were acquired [33]. Thirty cardiac phases were obtained per heartbeat. Biventricular end systolic and end diastolic volumes (ESV and EDV, respectively) and ejection fractions (EF) and the LV mass index were quantitatively evaluated in a standard way from the short-axis stack. Left and right atrial areas were measured from the 4-chamber view projection in the ventricular end-systolic phase. Biventricular volumes, LV mass, and atrial areas were normalized for the body surface area. 

For the assessment of replacement/focal myocardial fibrosis, late gadolinium-enhancement (LGE) short-axis, vertical, horizontal, and oblique long-axis images were collected 10–18 min after the intravenous injection of Gadobutrol (Gadovist^®^; Bayer Schering Pharma, Berlin, Germany) at the dose of 0.2 mmoL/kg of body weight, using a fast gradient-echo inversion recovery sequence. LGE imaging was not performed in patients with a glomerular filtration rate < 30 mL/min/1.73 m^2^ and in patients who declined the contrast medium administration. The extent of LGE was determined semi-quantitatively by counting the number of LV segments exhibiting visually determined LGE. Enhancement was considered present when visualized in two different views [34].

### 2.4. Diagnostic Criteria

OXY-Adsorbent (OXY) serum levels higher than 350 μmol HClO/mL were considered normal (manufacturer’s indications). The expected d-ROM levels in healthy individual are between 250 and 320 AU, while higher values denote a surplus of peroxides indicative of a systemic increase in ROS levels (manufacturer’s indications). The applied reference ranges are reported in Table 1.

A MRI LIC ≥ 3 mg/g dry weight (dw) indicated significant hepatic iron load [35]. The highest threshold of normal global pancreas R2* value was 38 Hz [36]. The value of 50 Hz (T2* = 20 ms) was used as a “conservative” normal value for segmental and global heart R2* values [37].

Normal glucose tolerance (NGT) was defined as fasting plasma glucose (FPG) < 100 mg/dL and 2 h glucose < 140 mg/dL. Impaired fasting glucose (IFG) was defined by FPG levels between 100 and 126 mg/dL. Impaired glucose tolerance (IGT) was diagnosed when FPG was less than 126 mg/dL, and 2 h plasma glucose ranged between 140 and 200 mg/dL. Diabetes mellitus (DM) was defined by FPG ≥ 126 mg/dL or 2 h plasma glucose ≥ 200 mg/dL during an OGTT or a random plasma glucose ≥ 200 mg/dL with classic symptoms of hyperglycaemia or hyperglycaemic crisis [38].

Cardiac involvement was defined as the presence of heart failure (HF) and arrhythmias. Heart failure was diagnosed by expert cardiologists based on medical history, physical examination, imaging findings, and laboratory test results, according to the AHA/ACC guidelines [39]. Arrhythmias were diagnosed according to the manifestations of electrocardiogram (ECG) and Holter monitoring, and were classified according to the AHA/ACC Guidelines [40].

Based on the agreement between the self-reported and prescribed chelation regimen, compliance was defined excellent (>80%), good (60–80%), and insufficient (<60%). 

### 2.5. Statistical Analysis

SPSS version 27.0 (IBM Corp, Armonk, NY, USA) and MedCalc version 19.8 (MedCalc Software Ltd., Ostend, Belgium) statistical packages were employed for statistical data analysis.

Continuous variables were presented as mean ± standard deviation (SD), while categorical variables were described using frequencies and percentages.

The Kolmogorov–Smirnov test was used to verify the normal distribution of quantitative variables.

Correlation analysis was conducted employing Pearson’s or Spearman’s tests where appropriate.

The comparison between two groups was carried out using the independent-samples *t*-test for continuous variables that followed a normal distribution, the Wilcoxon’s signed rank test for continuous values with non-normal distribution, and χ^2^ testing for categorical variables.

Analysis of covariance (ANCOVA) was employed to assess the influence of covariates on group differences in continuous parameters. Covariates were included if proved significantly different between groups and correlated with the outcome being assessed. When necessary, outcomes were log-transformed to normalize the residual distributions and to equalize the residual variance.

The level of statistical significance was set at a 2-tailed probability value ≤ 0.05.

## 3. Results

### 3.1. Patient’s Characteristics 

All the 58 β-TDT patients were white and had a mean age of 37.55 ± 7.83 years (range: 18–55 years). Genders were well balanced, with 51.7% women and 48.3% men. All patients received regular blood transfusions since early childhood to maintain a mean pre-transfusion hemoglobin of 9–10 g/dL and were undergoing chelation therapy. 

Demographic, clinical, laboratory, and instrumental characteristics of the β-TDT patients are summarized in Table 2 and Table 3.

**Figure 1:**
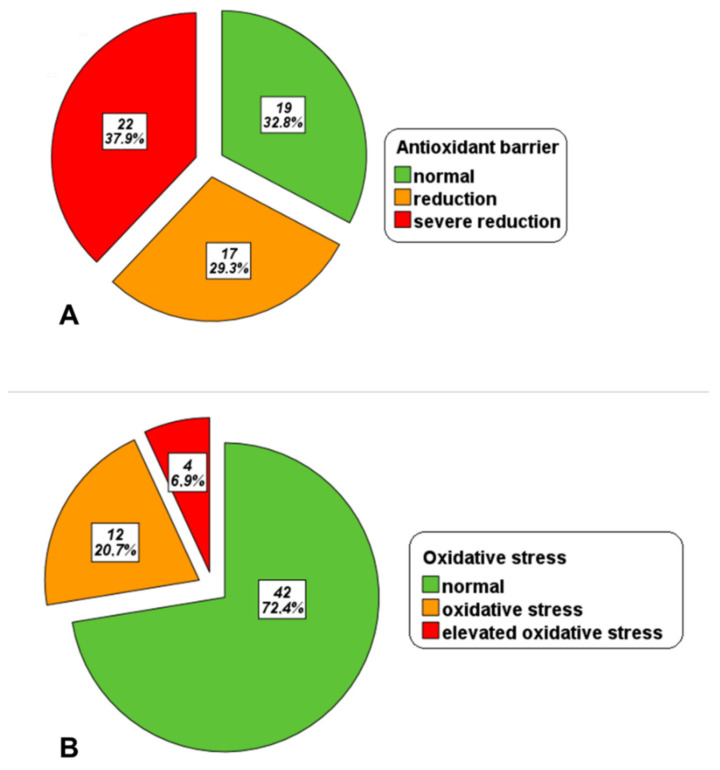
Distribution of β-TDT patients in the groups identified based on the OXY levels (**A**) and d-ROM values (**B**).

The mean level of OXY in the whole patient population was 323.75 ± 113.19 μmol HClO/mL. OXY levels were normal in 19 (32.8%) patients, reduced in 17 (29.3%) patients, and severely reduced in 22 (37.9%) patients (Figure 1A). 

The mean level of d-ROMs in serum was 305.12 ± 62.19 AU. In total, 42 (72.4%) patients showed normal d-ROMs values, 12 (20.7%) showed oxidative stress, and 4 (6.9%) showed elevated oxidative stress (Figure 1B).

No association was detected between OXY and d-ROM levels (R = −0.014; *p* = 0.917). In fact, the frequency of patients with increased d-ROM levels was comparable between patients with normal and reduced OXY (21.1% vs. 30.8%; *p* = 0.541).

### 3.2. Correlation of Oxidative Stress-Related Biomarkers with Demographic, Clinical and Biochemical Parameters

Table 2 shows the correlation of oxidative balance markers with demographic, clinical, and biochemical parameters.

OXY levels showed no link with gender, age, age at start of regular transfusions, chelation starting age, presence of splenectomy, and any biochemical parameter.

D-ROMs did not differ between β-TDT males and females or between splenectomized and non-splenectomized patients, but showed a significant positive association with aging. D-ROM levels were significantly correlated with fasting glycemia and NT-proBNP levels. No further relationship among the biochemical parameters examined was observed.

### 3.3. Correlation of Oxidative Stress-Related Biomarkers with Cardiovascular Magnetic Resonance Parameters

**Figure 2:**
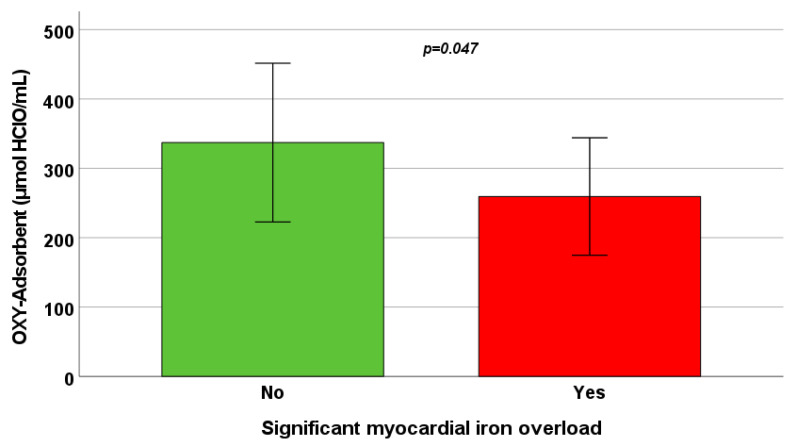
Mean OXY levels in patients without and with significant myocardial iron overload. The bars in the boxes represent the SD.

OXY levels were not associated with hepatic or pancreatic iron levels, but showed a negative correlation with global heart R2* values and with the number of segments with R2* > 50 Hz (Table 3). Patients with significant myocardial iron overload (10/58 = 17.2%) had significant lower OXY levels than patients without significant myocardial iron overload (259.42 ± 84.66 μmol HClO/mL vs. 337.16 ± 114.47 μmol HClO/mL; *p* = 0.047) (Figure 2).

No association was detected between d-ROM levels and hepatic, pancreatic, and cardiac iron levels (Table 3).

OXY levels and d-ROMs were comparable between β-TDT patients without and with replacement myocardial fibrosis, and were uncorrelated with all biventricular function parameters and with atrial dimensions.

### 3.4. Oxidative Status and Complications

**Figure 3:**
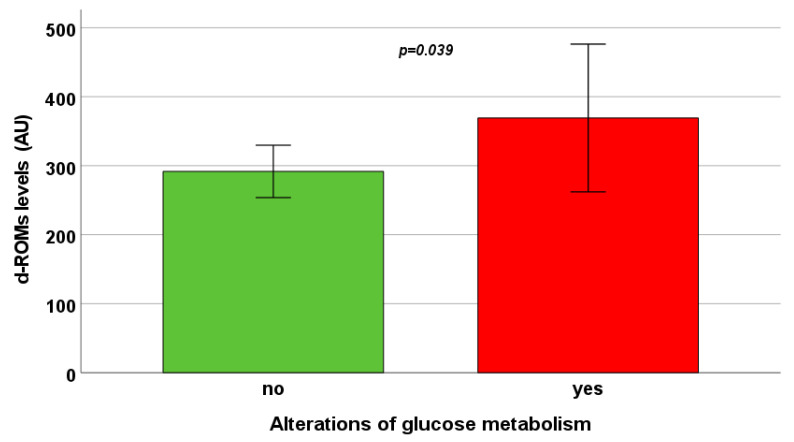
Mean d-ROMs levels in patients with normal and altered glucose metabolism. The bars in the boxes represent the SD.

Ten (17.2%) β-TDT patients had an altered glucose metabolism: two IFG, one IGT, and seven DM. No difference between patients with normal and altered glucose metabolism was found in terms of OXY levels (327.25 ± 114.75 μmol HClO/mL vs. 306.97 ± 109.57 μmol HClO/mL; *p* = 0.621), while patients with an altered glucose metabolism showed increased d-ROMs levels (369.19 ± 107.05 AU vs. 291.77 ± 37.97 AU; *p* = 0.039) (Figure 3). The difference in d-ROM levels remained significant after the correction for age (*p* = 0.010).

**Figure 4:**
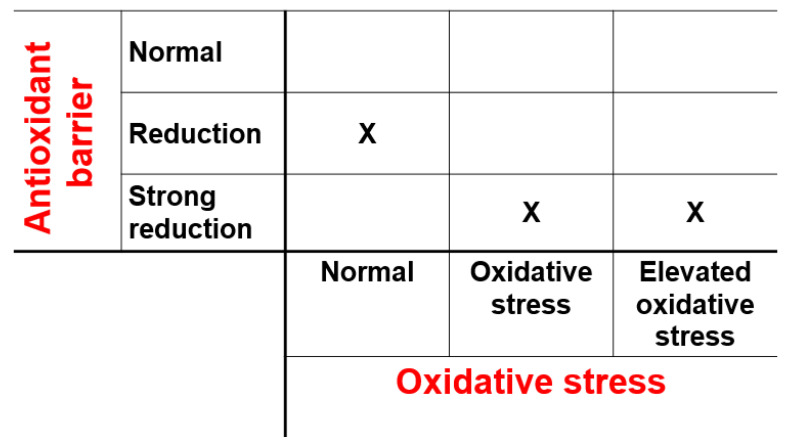
Distribution of oxidative stress markers in the three patients (represented by the X) with cardiac complications.

Three (5.2%) patients had a cardiac complication: one HF with persevered ejection fraction, and two arrhythmias (one atrial fibrillation and one hyperkinetic ventricular arrhythmia). All three patients with a cardiac complication had a reduced OXY value, and two of them had also in increased d-ROM level (Figure 4).

### 3.5. Oxidative Status and Chelation Therapy

Five groups of patients were identified based on the chelation regimen: five (8.6%) patients treated with desferrioxamine (DFO), twelve (20.7%) with deferiprone (DFP), twenty-nine (50.0%) with deferasirox (DFX), eight (13.8%) with combined DFO + DFP, and four (6.9%) with sequential DFO/DFP. 

The mean administered dosages of the chelators were: (1) DFO in monotherapy 36.67 ± 15.28 mg/kg body weight via subcutaneous route on 6.50 ± 0.58 days/week; (2) DFP in monotherapy 71.78 ± 11.72 mg/kg body weight with a frequency of 7 days/week; (3) DFX 28.74 ± 7.03 mg/kg body weight with a frequency of 7 days/week; (4) DFO in combined regimen 40.01 ± 6.46 mg/kg body weight per day with a frequency of 3.71 ± 1.11 days/week, and DFP in combined regimen 80.57 ± 16.55 mg/kg body weight per day with a frequency of 7 days/week; and (5) DFO in sequential regimen 40.00 mg/kg body weight per day with a frequency of 3 days/week, and DFP in sequential regimen 65.00 ± 13.23 mg/kg body weight per day with a frequency of 3.67 ± 0.58 days/week. Almost all patients (57/58 = 98.3%) had a good/excellent compliance (correspondence between taken and prescribed chelator ≥ 60%). 

**Figure 5:**
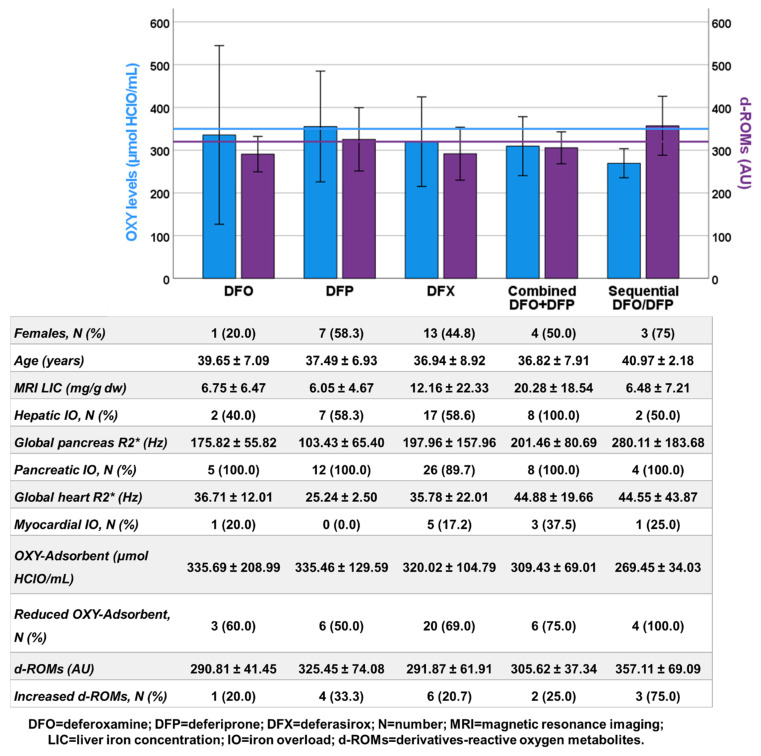
Characteristics of the five groups identified based on the chelation regimen. The horizontal lines represent the cut-off of normality for OXY-Adsorbent levels (purple line; 350 μmol HClO/mL) and d-ROMs levels (blue line; 320 AU)).

Figure 5 shows the mean OXY and d-ROM levels in the five treatment groups. Due to the low number of patients in some treatment groups, no statistical comparison among the five groups was performed. 

Compared to patients who received iron chelator in monotherapy (DFO or DFP or DFX; N = 46), those who received two iron chelators simultaneously or on alternate days/week (N = 12) had lower OXY levels (296.10 ± 61.10 μmol HClO/mL vs. 330.97 ± 122.72 μmol HClO/mL; *p* = 0.420), higher d-ROMs levels (322.78 ± 53.21 AU vs. 300.51 ± 64.06 AU; *p* = 0.084), higher MRI LIC values (15.68 ± 16.71 mg/g dw vs. 9.98 ± 18.10 mg/g dw; *p* = 0.120), lower global pancreas T2* values (6.49 ± 5.81 ms vs. 10.99 ± 8.71 ms; *p* = 0.107), and lower global heart T2* values (29.11 ± 13.01 ms vs. 35.24 ± 10.14 ms; *p* = 0.158), but no difference was statistically significant.

## 4. Discussion

Repeated blood transfusions and hemolysis, which characterize TDT, induce iron overload, an oxidative stress status, and toxicity with multi-organ dysfunction in such patients. Thus, oxidative stress retains a significant role in the pathogenesis and development of the thalassemia disease and its complications. For the first time, we assessed OXY blood values (sample antioxidant barrier to the oxidant milieu) in TDT patients, observing that two-third of them showed reduced levels (more than one-third with severely lowered OXY). The other main findings of our study were the significant and inverse correlation of OXY levels with cardiac iron levels by MRI (global heart R2* and number of segments with R2* > 50 Hz) and the significant relationship between d-ROM levels and cardiometabolic risk factors, as aging, glycemia, and NT-proBNP.

Different studies have been conducted in order to evaluate the oxidant and antioxidant status in TDT. For it concerns the antioxidant status, available results are quite conflicting, as the estimation of the total antioxidant capacity using different methods (which are not totally equivalent) or single antioxidants may be found increased in TDT, and as such considered as a compensatory response to an increased oxidant status, or effectively reduced and restored (or partially restored) after antioxidant supplementation [41,42,43,44,45,46]. These controversial data may depend by different reasons: the biomarker evaluated, the sample (e.g., erythrocytes, peripheral blood mononuclear cells, plasma or serum), or even proximity to transfusion [47,48]. Moreover, mutual cooperation among the different antioxidants may better face the oxidant attack, and thus the overall antioxidant capacity, reflecting the cumulative effect of all antioxidants present in the blood, may likely give more relevant biological information on the antioxidant barrier action compared to that obtained by the assessment of an individual antioxidant component. Nonetheless, different total antioxidant assays (e.g., Erel method; FRAP: Ferric Reducing Antioxidant Potential; TEAC: Trolox Equivalent Antioxidant Capacity; TRAP: Total-radical Trapping Antioxidant Parameter) in serum do not equally cover all antioxidant processes; in fact, they may poorly correlate with each other, representing an additional value and complementary contributions in the assessment of the overall antioxidant blood status [49]. In any case, a recent meta-analysis (a total of 1351 subjects: 770 thalassemic and 581 controls, 15 case–control studies), aiming to evaluate the differences in the total antioxidant capacity between thalassemic patients and healthy individuals, ultimately concluded that the total antioxidant capacity was decreased in thalassemic patients versus healthy subjects, suggesting a depletion of the antioxidant defenses due to exposition to elevated and chronic oxidative stress [48].

Our finding about the significant reduction of OXY levels among patients with myocardial iron overload recalls a previous study where we observed that uric acid (a potent antioxidant) was reduced according to increased oxidative stress and cardiac iron overload in TDT patients [50]. Thus, the possibility of using circulating biomarkers as adjuvant tool to estimate the cardiac iron and the oxidant status is very attractive. MRI represents the elective strategy to assess iron overload in different organs, including the heart [37,51,52,53,54,55], but it is more demanding than a simple blood test and generally done annually or biannually. Nonetheless, more confirmatory data are clearly needed to assess accuracy, reproducibility, and reliability of these biomarkers in this clinical setting before they conquer a real clinical utility, especially for OXY, which is not a routine laboratory parameter.

Cardiac complications consequent to iron deposition are a major cause of death in TDT patients [56] and the depletion of OXY in all the three patients with cardiac complications was an interesting finding. So, levels of OXY merit to be further investigated in future and larger studies to assess if this biomarker may help to determine the risk of individual patients to develop cardiac complications.

Although we did not observe very high d-ROM levels in our patients, the association of d-ROMs with the cardiovascular risk factors in our patients confirm previous data obtained in other clinical settings. In fact, levels of d-ROMs are associated with aging in the general population [57,58]. Moreover, d-ROMs are elevated in type 2 diabetes patients respect to controls, and are correlated to trend of glycemia in type 2 diabetes [59,60].

One interesting result of the present study was the significant association between levels of d-ROMs and NT-proBNP, which is an indicator of ventricular wall stress and volume overload in heart failure and left ventricular dysfunction, and a gold standard biomarker for diagnosis and prognosis of heart failure [61,62,63]. Previous data reported that d-ROMs were related to presence, severity, and adverse prognosis in heart failure patients [64,65]. These relationships remain to be further confirmed in the TDT population, where d-ROMs might be helpful as an additive tool to stratify the cardiac risk level.

No clear relationship was observed between the oxidative stress-related biomarkers (OXY and d-ROMs) and chelation therapy. However, those patients who received two iron chelators simultaneously or on alternate days/weeks presented a trend towards lower OXY levels and higher d-ROM values. This result may reflect the fact that the patients who require a more aggressive chelation are those with greater tissue iron deposition, suggesting a relationship between oxidative stress and severity of the disease in terms of heavier iron burden rather than a real modulation of oxidative stress from chelating agents. 

### Limitations

This study has some limitations that need to be acknowledged. 

Due to the low number of patients in some groups categorized according to chelation regimens, the difference in the oxidative stress-related biomarkers between treatments was not found to be significant, and no definitive conclusions can be drawn. Nonetheless, the possibility of a relationship between chelation therapy and oxidative stress represents an interesting study hypothesis that deserves to be further tested.

This was a cross-sectional study and, therefore, causal relationships could not be determined. Further prospective studies involving a larger patient cohort should be conducted to validate our findings.

The d-ROM test requires the presence of iron ions. So, anemia, iron overload, and chelation therapy may potentially interfere with the measured levels [66].

Self-reported adherence may overestimate adherence attitude (lower sensitivity). 

## 5. Conclusions

A reduced antioxidant status is frequent in TDT patients, where OXY might serve as an additive biomarker to assess heart iron status, and its merits need to be further evaluated to confirm its transversal and prospective relationship with cardiac complications. Instead, d-ROM levels resulted associated with cardiovascular risk factors, thus they might be helpful as additive simple and easily available biomarker to assess the cardiovascular risk burden in these patients. To note, oxidative stress can be improved or restored with antioxidant strategies that may act at various levels. Efforts can be done in future to further verify the role of OXY and d-ROMs in TDT pathophysiology, assessing whether they can serve as risk biomarkers and/or targets of interventions aimed to achieve an optimal redox balance, improving quality of life and overall outcomes for patients. 

## Figures and Tables

**Table 1:** Reference values of OXY-Adsorbent and d-ROMs levels.

OXY-Adsorbent Levels (μmol HClO/mL)	Antioxidant Barrier
>350	Normal range
280–349	Reduction
<279	Severe reduction
**d-ROMs Levels (AU)**	**Oxidative Stress Levels**
250–320	Normal range
321–400	Oxidative stress
>401	Elevated oxidative stress

**Table 2:** Demographic, clinical, and biochemical correlates of oxidative stress markers.

		OXY-Adsorbent	d-ROMs
Categorical Variable			
	**Frequency, N (%)**	**Difference of Oxidative Stress Parameter between Two Groups (Absent vs. Present)**	
Female sex	28 (48.3)	324.52 ± 110.99 vs. 322.93 ± 117.55 μmol HClO/mL (*p* = 0.958)	293.21 ± 58.29 vs. 317.88 ± 64.75 AU (*p* = 0.125)
Splenectomy	33 (56.9)	335.35 ± 132.26 vs. 314.97 ± 97.56 μmol HClO/mL (*p* = 0.599)	299.18 ± 47.56 vs. 309.62 ± 71.74 AU (*p* = 0.510)
**Continuous Variables**			
	**Mean Value**	**Correlation (R, *p*-Value) with Oxidative Stress Parameter**	
Age (years)	37.55 ± 7.83 years	R = 0.053, *p* = 0.693	**R = 0.343, *p* = 0.008**
Age at start of regular transfusions	21.85 ± 14.71 months	R = 0.097, *p* = 0.510	R = 0.184, *p* = 0.212
Chelation starting age	3.60 ± 2.45 years	R = 0.150, *p* = 0.270	R = 0.139, *p* = 0.307
Pre-transfusion hemoglobin	9.59 ± 0.59 g/dL	R = 0.080, *p* = 0.556	R = 0.138, *p* = 0.307
Mean ferritin	1313.17 ± 1562.05 ng/mL	R = −0.089, *p* = 0.510	R = 0.041, *p* = 0.762
ALT	40.26 ± 34.79 U/L	R = 0.010, *p* = 0.942	R = 0.182, *p* = 0.187
AST	36.21 ± 26.19 U/L	R = 0.074, *p* = 0.594	R = 0.240, *p* = 0.078
Total cholesterol	116.90 ± 36.55 mg/dL	R = 0.158, *p* = 0.274	R = 0.252, *p* = 0.077
Triglycerides	103.77 ± 43.68 mg/dL	R = 0.213, *p* = 0.141	R = −0.077, *p* = 0.597
HDL cholesterol	40.79 ± 15.29 mg/dL	R = −0.104, *p* = 0.529	R = 0.307, *p* = 0.057
Fasting glycemia	94.42 ± 20.11 mg/dL	R = −0.185, *p* = 0.185	**R = 0.302, *p* = 0.028**
NT-proBNP	138.56 ± 166.78 pg/mL	R = 0.046, *p* = 0.798	**R = 0.480, *p* = 0.004**

d-ROMs = derivatives-reactive oxygen metabolites; N = number; HDL = high-density lipoprotein; NT-proBNP = N-terminal prohormone of brain natriuretic peptide. The significant results are in bold.

**Table 3:** MRI correlates of oxidative stress markers.

		OXY-Adsorbent	d-ROMs
Categorical Variable			
	Frequency, N (%)	Difference of Oxidative Stress Parameter between Two Groups (Absent vs. Present)	
LGE	23/49 (46.9)	345.10 ± 112.46 vs. 322.47 ± 125.61 μmol HClO/mL (*p* = 0.326)	287.02 ± 37.03 vs. 314.66 ± 70.87 AU (*p* = 0.331)
**Continuous Variables**			
	**Mean Value**	**Correlation (R, *p*-value) with Oxidative Stress Parameter**	
MRI LIC	11.16 ± 17.83 mg/g dw	R = −0.001, *p* = 0.993	R = 0.142, *p* = 0.288
Global pancreas R2*	182.64 ± 134.30 Hz	R = −0.124, *p* = 0.352	R = 0.054, *p* = 0.689
Global heart R2*	35.54 ± 20.94 Hz	**R = −0.339, *p* = 0.009**	R = −0.039, *p* = 0.769
Number of segments with R2* > 50 Hz	2.88 ± 5.48	**R = −0.278, *p* = 0.034**	R = 0.075, *p* = 0.573
LV end-diastolic volume index	88.86 ± 20.09 mL/m^2^	R = 0.074, *p* = 0.583	R = −0.184, *p* = 0.170
LV end-systolic volume index	32.44 ± 10.36 mL/m^2^	R = 0.044, *p* = 0.744	R = −0.088, *p* = 0.517
LV mass index	63.51 ± 14.53 g/m^2^	R = 0.010, *p* = 0.943	R = −0.030, *p* = 0.826
LV ejection fraction	63.96 ± 6.26%	R = −0.035, *p* = 0.798	R = −0.034, *p* = 0.800
RV end-diastolic volume index	89.33 ± 20.79 mL/m^2^	R = 0.069, *p* = 0.610	R = −0.225, *p* = 0.092
RV end-systolic volume index	34.51 ± 11.74 mL/m^2^	R = 0.088, *p* = 0.517	R = −0.195, *p* = 0.145
RV ejection fraction	61.05 ± 6.16%	R = −0.048, *p* = 0.723	R = 0.210, *p* = 0.117
Left atrial area index	14.25 ± 3.27 cm^2^/m^2^	R = −0.067, *p* = 0.631	R = 0.208, *p* = 0.131
Right atrial area index	12.79 ± 1.92 cm^2^/m^2^	R = 0.085, *p* = 0.541	R = −0.146, *p* = 0.292

d-ROMs = derivatives-reactive oxygen metabolites; N = number; LGE = late gadolinium enhancement; MRI = magnetic resonance imaging; LIC = liver iron concentration; LV = left ventricular; RV = right ventricular. The significant results are in bold.

## Data Availability

The data presented in this study are available on request from the corresponding author. The data are not publicly available due to privacy.
